# RELIABILITY AND VALIDITY OF THE MCDR FOR HISPANIC OLDER ADULTS

**DOI:** 10.1093/geroni/igad104.1950

**Published:** 2023-12-21

**Authors:** Lisandra Mendoza, Patricia Garcia, Dilianna Padron, Andres Duarte, Michael Marsiske, Jacob Fiala, Ranjan Duara, Miriam Rodriguez

**Affiliations:** Bay Pines VA Health System, Bay Pines, Florida, United States; Indiana University School of Medicine, Indianapolis, Indiana, United States; Central Virginia VA Healthcare System, Richmond, Virginia, United States; Albizu University, Doral, Florida, United States; University of Florida, Gainesville, Florida, United States; University of Florida, Gainesville, Florida, United States; Mount Sinai Medical Center, Miami Beach, Miami Beach, Florida, United States; Indiana University Bloomington, Bloomington, Indiana, United States

## Abstract

**Objective:**

Effective functional measures can be used among Hispanics to diagnose and evaluate functional dementia progression, especially in the prodromal stages when treatment can be most effective. The current study examined the reliability and validity of the modified version of the modified Clinical Dementia Rating Scale (mCDR) compared to the Alzheimer’s Disease Cooperative Study (ADCS)-MCI-ADL. These are quantitative severity measures of functional impairment in dementing conditions. The mCDR uses an itemized informant-based structured interview with multiple-choice responses and does not include cognitive testing (Duara et al., 2010).

**Methods:**

Functional measures were administered to 101 White non-Hispanic Cognitively Normal (CN, n=20) and MCI (n=81) and 159 Hispanic CN (n=32) and MCI (n=127) participants in the patient’s primary language (English or Spanish). Inter-rater reliability and convergent validity for these instruments were examined. Multiple regression analyses were conducted to determine if the administration’s language (English vs. Spanish) moderated the raters’ scores relationships.

**Results:**

The results revealed moderate-to-good mCDR inter-rater reliability [β =.73, F (40,40) = 6.24, p < .001], even when controlling for language (β = .72, p < .001; main effect and interaction were not significant). ADCS-MCI-ADL showed moderate-to-good inter-rater reliability [β = .71, F (40,36.6) = 6.34, p < .001] also, when controlling for language (β = .72, p < .001; main effect and interaction were not significant). The mCDR and the ADCS-MCI-ADL were correlated at -.75, suggesting a high convergent validity (56%).

**Conclusions:**

The m-CDR is a valid and reliable quantitative functional measure among Hispanic individuals.

